# Coffee and Caffeine in Depression: Symptom-Level Modulation and Challenges in Nutripsychiatric Interpretation

**DOI:** 10.3390/nu18071064

**Published:** 2026-03-26

**Authors:** Iwona Turkowska, Aleksandra M. Rogowska, Mariusz Konieczny, Przemysław Domaszewski

**Affiliations:** 1 Institute of Health Sciences, University of Opole, Katowicka 68, 45-060 Opole, Poland; przemyslaw.domaszewski@uni.opole.pl; 2 Institute of Psychology, University of Opole, 45-052 Opole, Poland; 3 Faculty of Physical Education and Physiotherapy, Opole University of Technology, 45-758 Opole, Poland

**Keywords:** sleep-wake regulation, fatigue, anxiety sensitivity, psychomotor slowing, withdrawal symptoms, arousal regulation

## Abstract

Background: Evidence on the relationship between coffee, caffeine and depression remains inconsistent. Observational studies often report inverse associations, whereas experimental findings indicate potential adverse effects on anxiety and sleep. As a psychostimulant, caffeine increases alertness and perceived energy and may transiently improve mood and task engagement. Objective: This narrative review aims to reinterpret existing evidence on coffee and caffeine in depression from a symptom-level perspective, with emphasis on sleep–wake regulation, anxiety sensitivity, habitual use and withdrawal-related phenomena. Methods: Human studies published between 2000 and 2025 were synthesised narratively based on their relevance to depressive symptoms, arousal regulation, sleep and behavioural patterns of caffeine use, rather than for pooled effect estimation. Results: Caffeine may transiently alleviate fatigue and psychomotor slowing, but these effects are context-dependent and frequently counterbalanced by sleep disruption, anxiety and withdrawal-related fluctuations. Inverse associations observed in cohort studies may partly reflect reverse causality, residual confounding and relief of withdrawal symptoms rather than true antidepressant effects. Conclusions: Coffee and caffeine should not be conceptualised as antidepressant interventions. Instead, they appear to act as context-dependent modulators of symptom expression in depression, particularly affecting fatigue, alertness and sleep–wake stability. Failure to account for dose, habitual use, withdrawal and individual vulnerability may lead to overinterpretation of epidemiological findings in nutripsychiatric research.

## 1. Introduction

Depression remains one of the leading causes of disability worldwide and continues to represent a major clinical and public health challenge [1]. Although pharmacological and psychotherapeutic treatments are effective for many patients, a substantial proportion experience only partial remission, persistent fatigue or cognitive slowing, and frequent relapses [2]. This has led to increasing interest in modifiable lifestyle exposures that might influence vulnerability to depression or shape the expression of its symptoms alongside standard treatment.

Within this context, dietary factors have received growing attention. Nutritional psychiatry, and more recently nutripsychiatry, attempts to integrate metabolic, behavioural and neurobiological perspectives on mental health [3]. Coffee and caffeine occupy a somewhat unusual position in this field. Unlike most dietary components discussed in psychiatric research, caffeine has rapid and noticeable psychophysiological effects. As a psychostimulant, it increases alertness, perceived energy and subjective vigour [4,5], and may transiently improve mood and task engagement [6,7]. These effects overlap with core domains of depressive symptomatology, including fatigue, psychomotor slowing and impaired concentration, which partly explains why coffee consumption is frequently discussed in relation to depression.

Long-term observational studies have repeatedly suggested that habitual coffee and caffeine consumption is associated with a lower risk of clinically diagnosed depression, particularly among women [8,9]. Such findings are often interpreted as indicating a protective or even antidepressant-like effect of caffeine. However, this interpretation remains problematic. Associations observed in cohort studies may reflect behavioural correlates of coffee consumption, such as structured daily routines or occupational engagement, rather than direct therapeutic effects of caffeine. In practice, higher coffee intake may co-occur with structured daily routines, occupational engagement and social activity, which are factors that are independently associated with better mental health and are often adjusted for in epidemiological studies [10,11].

It is also important to recognise caffeine not only as a dietary exposure but as a pharmacologically active compound with clinical applications. In psychiatry, caffeine has been used as an adjunct during electroconvulsive therapy to facilitate and prolong seizure duration in patients with severe depression, thereby increasing treatment efficacy [12]. This observation is sometimes cited as indirect support for the beneficial neurobiological effects of caffeine in depression. Still, such use occurs in a highly controlled medical context and should not be extrapolated to everyday coffee consumption without caution.

At the same time, a growing body of evidence indicates that excessive caffeine intake may have unfavourable psychological consequences [13]. Very high doses can induce anxiety, dysphoria and inner tension, which may worsen overall symptom burden rather than alleviate it [14]. Observational data also suggest that high levels of coffee consumption, often operationalised as five or more cups per day, can be associated with increased anxiety, frequently co-occurring with depressive symptoms [15]. Among young adult men, frequent use of energy drinks has likewise been linked to higher levels of depressive symptoms and stress, although these relationships are likely shaped by broader lifestyle and behavioural factors [1,16,17].

Another layer of complexity arises from patterns of self-medication. Patients experiencing lethargy, low energy or hypersomnia in the course of depression often consume large amounts of caffeine in an attempt to counteract these symptoms. In some cases, daily intake may exceed the recommended amount, suggesting that caffeine is used instrumentally to suppress fatigue rather than for hedonic reasons [18]. Such compensatory use can transiently increase alertness but may also lead to agitation combined with depressed mood, creating a mixed and clinically ambiguous effect profile.

Individual sensitivity further modifies these responses. Evidence suggests that patients with depression may display heightened vulnerability to the anxiogenic effects of caffeine compared with healthy individuals, which may limit its tolerability in clinical populations [19]. This variability highlights that caffeine cannot be assumed to exert uniform effects across individuals with depressive symptoms.

Withdrawal phenomena constitute an additional and often underestimated issue. Abrupt cessation of habitual caffeine intake frequently produces fatigue, irritability, reduced concentration and low mood, which are symptoms that overlap closely with core features of depression [20]. As a result, short-term mood improvements following caffeine consumption may partly reflect relief from withdrawal rather than genuine enhancement of baseline affective functioning. This distinction is rarely addressed in epidemiological or experimental studies and may contribute to overestimation of beneficial effects.

An additional aspect that is sometimes invoked in discussions on caffeine and depression concerns the anti-inflammatory and antioxidant properties of coffee as a complex dietary matrix. Coffee contains numerous bioactive compounds, including polyphenols such as chlorogenic acids, which have been shown in experimental and observational studies to exert antioxidant and anti-inflammatory effects at the systemic level [6]. This observation is often considered relevant in light of inflammatory theories of depression, which propose that low-grade chronic inflammation may contribute to symptom persistence in at least a subset of patients. However, the relevance of these mechanisms for depressive symptoms remains uncertain, with most evidence linking coffee consumption to inflammatory markers derived from general population studies rather than clinically characterised depressive samples [21,22]. Moreover, any potential anti-inflammatory effects of coffee are likely to be modest, cumulative and strongly dependent on overall dietary context, preparation methods and individual metabolic profiles. They are therefore unlikely to explain short-term changes in mood or energy that are more directly related to caffeine’s psychostimulant properties. Consequently, while the anti-inflammatory and antioxidant properties of coffee may represent a biologically plausible background pathway within the inflammatory framework of depression, they should not be interpreted as evidence of a direct antidepressant effect. A more cautious interpretation is that coffee may contribute to long-term modulation of physiological vulnerability, rather than exerting clinically meaningful improvements in depressive symptomatology on its own.

**Figure 1:**
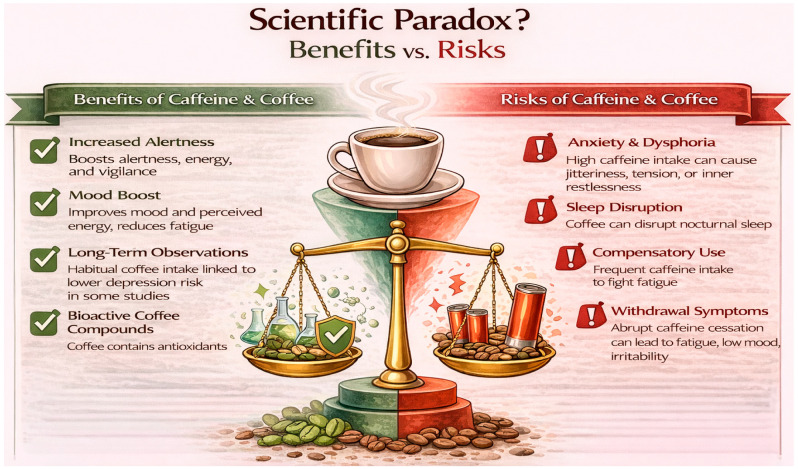
Scientific paradox of benefits and risks regarding coffee and caffeine.

Taken together, the existing literature presents a paradoxical picture, as shown in Figure 1. Existing studies report both short-term improvements in alertness and potential worsening of sleep and anxiety, indicating that caffeine may produce opposing effects across symptom domains. These opposing processes are rarely captured when depression is treated as a binary diagnostic outcome rather than a heterogeneous constellation of symptoms. A dichotomous case–control framing may therefore obscure symptom-specific effects, particularly when an exposure such as caffeine simultaneously improves energy-related symptoms while worsening anxiety or sleep-related burden. This limitation is consistent with a growing body of research suggesting that depression is a heterogeneous construct composed of partially distinct symptom dimensions, which may differ in their underlying mechanisms, functional consequences and responsiveness to interventions [23,24]. Symptom-level approaches have therefore been proposed as a more informative framework for understanding both aetiology and treatment effects, particularly in the context of exposures that may differentially influence energy, sleep and affective regulation [25]. For this reason, the present review adopts a symptom-level perspective and focuses on key mediating and moderating pathways, including sleep–wake regulation, anxiety sensitivity, habitual use patterns and withdrawal dynamics. Rather than estimating pooled risk effects, the aim is to clarify how coffee and caffeine should be interpreted within a nutripsychiatric framework that integrates epidemiological observations with underlying physiological and behavioural mechanisms. This framework allows consideration of mechanisms that may bias epidemiological findings, including reverse causality, withdrawal-related symptom relief and unmeasured sleep disturbance.

## 2. Methods

This review was conducted as a narrative and interpretative synthesis aimed at examining how coffee and caffeine relate to depressive symptom expression and its behavioural and physiological moderators. The objective was not to provide pooled quantitative estimates but to critically integrate epidemiological, experimental and mechanistic evidence within a symptom-level framework. This approach was chosen because most existing meta-analyses focus primarily on binary diagnostic outcomes and offer limited insight into symptom-specific pathways, sleep–wake regulation and patterns of habitual caffeine use. In addition, substantial heterogeneity in exposure definitions, including dose, timing and habitual use, as well as variability in outcome measures and study designs, limits the interpretability of pooled estimates at the symptom level. For this reason, a narrative, mechanism-oriented synthesis was considered more appropriate for the aims of the present review. This review was not intended to evaluate adherence to a defined dietary pattern, but rather to examine coffee and caffeine as discrete psychoactive exposures within a nutripsychiatric context. Accordingly, the composition of dietary patterns or specific food groups associated with coffee consumption was not analysed. A structured literature search was performed in PubMed, Scopus and Web of Science to identify human studies published between January 2000 and December 2025. The lower time limit was chosen to focus on studies conducted within contemporary diagnostic and methodological frameworks, ensuring greater comparability of findings. In total, approximately 60 studies were considered in the narrative synthesis, including epidemiological, experimental and mechanistic research. The search combined terms related to caffeine exposure (“caffeine”, “coffee”, and “energy drinks”) with terms referring to depression and related constructs (“depression”, “major depressive disorder”, “depressive symptoms”, “fatigue”, “sleep”, “anxiety”, and “arousal”). Searches were supplemented by screening reference lists of relevant reviews and key primary studies to ensure coverage of the influential epidemiological and experimental literature. Studies were considered eligible based on the following inclusion criteria: (1) prospective cohort or case–control studies examining associations between coffee or caffeine intake and depression or depressive symptoms; (2) meta-analyses or Mendelian randomization studies addressing causal inference between caffeine exposure and depressive outcomes; (3) controlled human experimental studies investigating effects of caffeine on mood, alertness, fatigue, anxiety or sleep; and (4) mechanistic human research directly relevant to proposed pathways (e.g., adenosine signalling and sleep disruption) with clear implications for depressive symptom expression. Exclusion criteria included studies focusing exclusively on unrelated psychiatric conditions or on non-human models, unless they provided essential mechanistic context not available in human research. When multiple publications addressed similar datasets or outcomes, priority was given to larger, more recent or methodologically stronger studies. Selection was based on conceptual relevance rather than exhaustive inclusion of all available studies.

Given the substantial heterogeneity in study designs, populations and outcome definitions, a formal quantitative synthesis was not considered appropriate. Instead, the literature was synthesised narratively, with emphasis on identifying consistent and inconsistent patterns across epidemiological, experimental and mechanistic evidence.

The synthesis was organised around predefined domains reflecting the conceptual framework of the review, including neurobiological mechanisms, sleep–wake regulation, behavioural patterns of use and individual vulnerability. Within each domain, findings were interpreted in relation to their direction, consistency and methodological strength. Greater weight was given to studies with clearer temporal structure, experimental control or direct relevance to symptom-level outcomes. Particular attention was paid to potential sources of bias, including reverse causality, residual confounding, withdrawal-related symptom reversal and unmeasured sleep disturbance. In addition, the interpretation of findings was guided by whether the observed effects were more consistent with short-term modulation of arousal-related symptoms or with longer-term influence on depressive disorder processes.

The synthesis was guided by a predefined conceptual framework distinguishing between: (a) acute, state-dependent modulation of symptoms such as fatigue, alertness and psychomotor activation; and (b) potential longer-term effects on the course and risk of depressive disorders. This framework is based on the assumption that caffeine primarily influences momentary symptom expression through modulation of arousal and behavioural regulation, rather than exerting direct effects at the level of depressive disorder as a diagnostic entity. This distinction was used to evaluate whether reported benefits reflect genuine modification of depressive pathology or primarily transient changes in arousal and energy regulation.

The thematic sections that follow were organised to reflect this framework by grouping the literature into key mediating and moderating domains most relevant to symptom-level interpretation, including neurobiological mechanisms of arousal, sleep–wake regulation, patterns of habitual use and withdrawal, as well as individual vulnerability profiles.

## 3. Caffeine, Adenosine Signalling and Psychomotor Activation

**Figure 2:**
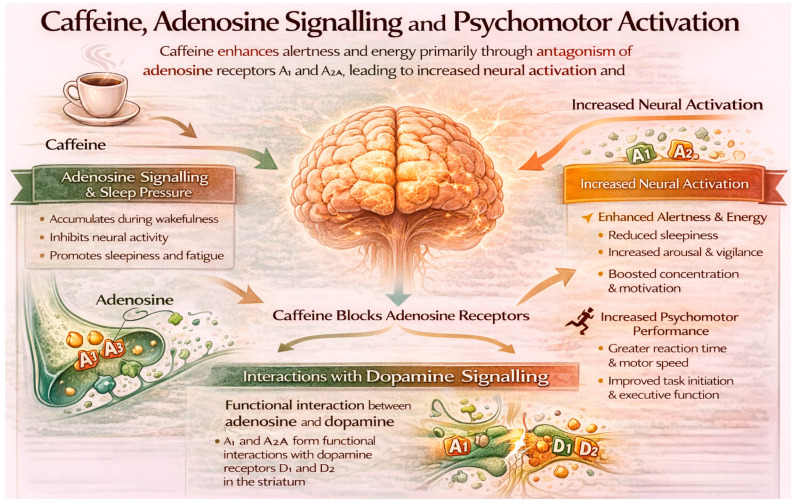
Neurobiological mechanism between adenosine, caffeine and its effect on the brain.

The primary neurobiological mechanism through which caffeine influences psychological functioning is antagonism of adenosine receptors, particularly A_1_ and A_2_A receptors in the central nervous system [17]. Adenosine acts as an inhibitory neuromodulator that accumulates during wakefulness and contributes to sleep pressure, reduced alertness and psychomotor slowing [26]. As it is illustrated in Figure 2, by blocking adenosine signalling, caffeine produces a state of increased neural activation subjectively experienced as greater energy, alertness and readiness to engage in cognitive and behavioural tasks [27,28].

This mechanism is directly relevant to fatigue and psychomotor slowing, which are among the most persistent symptoms of depressive episodes. Fatigue, low energy and psychomotor slowing are among the most persistent and disabling features of depressive episodes and often remain even when mood symptoms partially improve. Caffeine-induced adenosine antagonism can transiently counteract these symptoms, leading to short-term improvements in perceived energy and task initiation. Experimental and psychopharmacological studies consistently show that caffeine enhances alertness and performance primarily under conditions of low baseline arousal, which is a state commonly observed in depression-related fatigue [27,29].

At the same time, these effects are inherently state-dependent rather than disease-modifying. The pharmacological action of caffeine operates on momentary arousal regulation and does not directly target core pathophysiological processes implicated in depressive disorders. Consequently, improvements in energy or concentration following caffeine intake should be interpreted as an acute modulation of symptom expression rather than evidence of an antidepressant effect at the level of disorder trajectory. This distinction is consistent with broader psychopharmacological evidence indicating that caffeine primarily alters vigilance and perceived effort rather than underlying mood disorder mechanisms [30].

A further layer of complexity arises from interactions between adenosine and dopaminergic signalling. Adenosine receptors form functional interactions with dopamine receptors within striatal circuits involved in motivation and psychomotor activation. Through this pathway, caffeine may indirectly enhance dopaminergic tone, which could plausibly influence anhedonia-related processes and behavioural activation [28]. However, direct clinical evidence linking this mechanism to sustained improvements in depressive symptoms remains limited, and the magnitude of such effects in real-world settings is likely modest.

Importantly, the same mechanism that reduces fatigue can also increase physiological and subjective arousal beyond an optimal range. When baseline arousal is low, as is common in depression characterised by anergia and hypersomnia, caffeine may restore a more functional level of activation [31]. When baseline arousal is already elevated, or when anxiety symptoms are prominent, the identical pharmacological action may lead to overstimulation, restlessness and inner tension [15].

Temporal dynamics further constrain interpretation. The effects of adenosine antagonism are relatively short-lived [17], whereas sleep–wake regulation depends on cumulative homeostatic and circadian processes. Repeated or late-day caffeine intake may delay sleep onset and fragment sleep architecture, thereby worsening next-day fatigue and mood regulation [32]. In such cases, the apparent short-term benefit in alertness may be offset by longer-term destabilisation of sleep and energy rhythms, creating a pattern of fluctuating rather than sustained improvement.

Adenosine receptor antagonism primarily produces short-lived increases in alertness and energy, with effects that depend on baseline arousal and sleep vulnerability. Its effects are primarily acute, state-dependent and strongly moderated by baseline arousal, anxiety sensitivity and sleep vulnerability. This perspective supports the interpretation of caffeine as a context-dependent functional modulator rather than a direct therapeutic agent in depression.

## 4. Sleep–Wake Regulation as a Central Mediator

Sleep disturbance is a core feature of depression and one of the most robust predictors of symptom persistence and relapse. Insomnia, fragmented sleep and circadian misalignment are common across depressive phenotypes and often precede the onset or worsening of mood symptoms [32]. Within this context, sleep–wake regulation represents a central pathway through which coffee and caffeine may influence depressive symptom expression, not as a direct antidepressant mechanism, but as a mediator shaping daily functioning and energy balance.

Caffeine exerts its primary effects on sleep through antagonism of adenosine receptors, delaying sleep onset, reducing total sleep time and increasing sleep fragmentation [33]. Controlled experimental studies have demonstrated that caffeine consumed even several hours before bedtime can significantly impair sleep continuity and duration [33,34]. These effects are not trivial, as sleep disruption is closely linked to worsening mood regulation, cognitive performance and emotional reactivity, all of which are highly relevant to depressive symptomatology [31].

At the same time, improved daytime alertness following coffee consumption may temporarily counteract sleep-related fatigue [17]. For individuals experiencing hypersomnia, excessive daytime sleepiness or marked psychomotor slowing, caffeine can facilitate engagement in daily tasks and social interaction. This short-term functional benefit is clinically meaningful, but it may coexist with deterioration in nocturnal sleep quality. As a result, caffeine use can create a feedback loop in which it is consumed to offset fatigue that is partly maintained by prior sleep disruption.

Timing of intake is therefore a critical, yet often insufficiently measured, moderator. Morning consumption may enhance alertness with relatively limited impact on subsequent sleep in many individuals, whereas afternoon or evening intake is more likely to interfere with sleep initiation and circadian alignment [32]. Large epidemiological studies rarely capture the detailed timing of caffeine exposure, which limits the ability to distinguish adaptive patterns of use from those that are potentially destabilising.

Inter-individual variability further complicates interpretation. Sensitivity to caffeine’s sleep-disrupting effects varies widely and is influenced by genetic factors, habitual intake, age and baseline sleep quality [5,6]. Individuals with depression may be particularly vulnerable to these effects, even when they do not subjectively attribute sleep problems to caffeine. This discrepancy between perceived tolerance and physiological sleep disruption may contribute to the underestimation of caffeine-related sleep effects in self-report studies.

From a symptom-level perspective, sleep–wake regulation should be viewed as a mediator rather than a secondary outcome. Improvements in daytime energy or concentration following caffeine intake do not necessarily indicate beneficial effects on depressive pathology if they are accompanied by deterioration in sleep continuity. Conversely, reductions in caffeine intake may initially worsen daytime fatigue due to withdrawal or reduced stimulation, but over time, may contribute to more stable sleep patterns and potentially more consistent mood regulation.

Taken together, the interaction between caffeine and sleep–wake regulation illustrates why population-level associations between coffee consumption and depression are difficult to interpret. Depending on timing, individual sensitivity and baseline sleep vulnerability, caffeine may either support or undermine processes that are central to mood regulation. Ignoring sleep as a mediating pathway risks conflating short-term increases in alertness with longer-term effects on depressive symptom burden.

## 5. Tolerance, Habituation and Withdrawal: Adaptive Use or Symptom Masking?

Caffeine consumption is rarely an isolated exposure. For most individuals, including those with depressive symptoms, coffee intake is habitual and embedded in daily routines. As a result, tolerance, habituation and withdrawal represent central mechanisms shaping the psychological effects attributed to caffeine, yet they can often be insufficiently considered in depression research.

With repeated exposure, partial tolerance develops to many of caffeine’s acute effects, particularly those related to alertness and psychomotor activation. This adaptation is linked to neurobiological changes within adenosine signalling pathways, including upregulation of adenosine receptors, which reduces the subjective intensity of stimulation over time [27]. Importantly, tolerance is incomplete and uneven. While some stimulating effects attenuate, other outcomes, such as sleep disruption, may persist despite perceived habituation, creating a mismatch between subjective tolerance and physiological impact.

Withdrawal represents the inverse aspect of this adaptation process. In regular caffeine users, even short periods of abstinence can produce fatigue, low mood, irritability and reduced concentration [35]. These symptoms overlap closely with core depressive features, including anergia and cognitive slowing, which complicates the interpretation of both observational and experimental findings [20]. Consequently, resumption of caffeine intake may alleviate withdrawal-related discomfort and be perceived as mood improvement, even when overall functioning does not exceed the individual’s non-caffeinated baseline.

This dynamic has important implications for interpreting epidemiological associations. Individuals with higher habitual coffee intake may appear to have better mood or lower depression risk, partly because regular consumption prevents withdrawal-related symptoms [36]. Experimental studies showing short-term improvements after caffeine administration may likewise reflect withdrawal reversal rather than genuine enhancement of underlying affective state, a possibility that has been repeatedly emphasised in the psychopharmacological literature [30].

From a behavioural perspective, caffeine use in depression may function as an adaptive self-regulation strategy. Individuals experiencing fatigue, low motivation or cognitive slowing may rely on coffee to maintain daily functioning, especially in occupational or socially demanding contexts. Such use can be functional in the short term, supporting task initiation and engagement, but it may also obscure the underlying severity of symptoms and delay recognition of contributing factors such as poor sleep or comorbid anxiety.

The interaction between tolerance and sleep further complicates this picture. As tolerance develops to daytime stimulation, individuals may increase the dose or shift consumption later in the day to maintain alertness [34]. This pattern can inadvertently increase sleep disruption, which then amplifies next-day fatigue and reinforces further caffeine use. Over time, this cycle may contribute to greater symptom instability rather than sustained improvement, even if subjective reliance on caffeine is experienced as helpful.

These mechanisms suggest that caffeine may reduce the perceived intensity of fatigue without necessarily changing the underlying severity of depressive symptoms. Caffeine may reduce the perceived burden of certain depressive features, particularly fatigue and psychomotor slowing, without necessarily altering the underlying course of the disorder. In some individuals, habitual use may even contribute indirectly to symptom persistence through sleep disruption or heightened physiological arousal. Within a nutripsychiatric framework, tolerance and withdrawal should therefore be viewed as key moderators rather than peripheral side effects. Understanding how individuals with depression use caffeine across days and weeks, rather than in isolated doses, is essential for interpreting observed associations and avoiding overly simplistic conclusions about benefit or harm.

## 6. Inflammation, Oxidative Stress and the Coffee Matrix: Indirect and Context-Dependent Pathways

Low-grade chronic inflammation and oxidative stress are increasingly recognised as central, interrelated mechanisms in the aetiology and progression of many non-communicable diseases, including metabolic, cardiovascular and neuropsychiatric disorders [37]. These processes reflect long-term dysregulation of immune and metabolic homeostasis shaped by lifestyle, environmental exposures and individual biological vulnerability. In recent years, similar mechanisms have been proposed as relevant to the pathophysiology of depression, particularly in subgroups characterised by fatigue, anhedonia and reduced psychomotor activity [38].

Within this broader framework, coffee and its bioactive constituents have attracted interest as potential modulators of inflammatory and oxidative processes. Coffee is a complex dietary exposure containing numerous compounds beyond caffeine, including chlorogenic acids, caffeic acid, diterpenes, trigonelline and melanoidins formed during roasting [6]. Many of these substances exhibit antioxidant and anti-inflammatory properties in experimental and observational research [39]. Observational studies have also linked habitual coffee consumption with lower levels of certain inflammatory markers and improved metabolic profiles, although these associations are modest and context-dependent [40].

From the perspective of inflammatory theories of depression, such properties may appear conceptually relevant. If low-grade chronic inflammation contributes to symptom persistence in some patients, long-term dietary exposures with anti-inflammatory potential could theoretically modify physiological vulnerability [38,40]. However, direct evidence linking coffee consumption to reductions in depressive symptoms through inflammatory pathways remains limited. Most studies examining coffee and inflammation have been conducted in general population samples rather than clinically characterised depressive cohorts, which restricts direct translational inference.

Importantly, inflammation-related mechanisms are unlikely to explain the immediate psychological effects of coffee that are typically reported by users, such as increased alertness or reduced fatigue. These short-term changes are more plausibly mediated by adenosine antagonism and arousal-related processes than by gradual modulation of inflammatory tone. Consequently, any potential anti-inflammatory benefits of coffee would be expected to operate over longer time scales and interact with broader dietary and metabolic context rather than produce rapid mood improvements.

Oxidative stress represents a related but distinct pathway. Experimental studies indicate that coffee consumption can increase total antioxidant capacity, largely due to its polyphenolic content [39]. In depression, oxidative stress has been associated with symptom severity and cognitive impairment, yet causal relationships remain uncertain [38]. Therefore, while the antioxidant properties of coffee are biologically plausible, they do not provide strong evidence for clinically meaningful antidepressant effects.

An additional layer of complexity concerns the interaction between coffee constituents and the gut microbiota. Polyphenols and melanoidins are partially metabolised by colonic bacteria, generating bioactive metabolites that may influence immune and metabolic signalling [6]. Alterations in gut microbiota composition have been implicated in depression through immune, neuroendocrine and metabolic pathways [41], but direct evidence linking coffee-induced microbiota changes to improvement in depressive symptoms is still sparse and largely indirect.

Taken together, inflammation- and redox-related mechanisms may constitute background pathways through which coffee consumption interacts with long-term physiological vulnerability to depression. However, these effects are likely modest, cumulative and highly dependent on individual metabolic and dietary context. Framing coffee primarily as an anti-inflammatory or antioxidant intervention for depression would therefore overstate the strength and specificity of current evidence. A more cautious interpretation is that the coffee matrix may contribute to subtle modulation of systemic processes that indirectly relate to depressive symptom expression, without exerting a direct or robust antidepressant effect.

## 7. Individual Vulnerability Profiles and Boundary Conditions

Responses to coffee and caffeine in depression are highly heterogeneous and shaped by individual vulnerability profiles. Rather than exerting uniform effects, caffeine interacts with pre-existing traits and comorbid symptoms that influence arousal regulation, sleep stability and affective reactivity [9,28]. Recognising these boundary conditions is essential for interpreting epidemiological associations, which typically report average effects that may obscure clinically meaningful inter-individual differences.

Anxiety-related vulnerability represents one of the most important moderators. Anxiety symptoms frequently co-occur with depression and can substantially alter responses to caffeine. Individuals with heightened anxiety sensitivity or prominent somatic anxiety are more likely to experience caffeine-induced nervousness, inner tension and autonomic arousal [42]. In such cases, stimulation that might reduce fatigue can simultaneously exacerbate anxiety-related distress, potentially worsening overall symptom burden. Experimental evidence indicates that patients with panic disorder and depressive symptoms may show stronger anxiogenic responses to caffeine than healthy controls, suggesting that identical pharmacological effects may shift arousal beyond an optimal range in vulnerable individuals [43].

Mood instability represents another relevant boundary condition. Mood disorders exist on a spectrum, and subthreshold bipolar features are not uncommon among individuals diagnosed with unipolar depression [44]. In this context, caffeine-related stimulation and sleep disruption may carry particular risks. Sleep loss and circadian instability are well-established triggers of mood destabilisation, and caffeine intake, especially in higher doses or later in the day, may indirectly contribute to such processes by delaying sleep onset and increasing fragmentation [32]. Although caffeine is not a causal factor in bipolar disorder itself, its interaction with sleep–wake regulation may be clinically relevant in individuals prone to mood fluctuations.

Sex differences and hormonal modulation further complicate interpretation. Caffeine metabolism is influenced by hormonal factors, including menstrual cycle phase, pregnancy and the use of hormonal contraception, which can prolong caffeine clearance and modify physiological responses [45]. Observational data also suggest sex-specific associations between caffeine intake and anxiety symptoms, indicating that women may, in certain contexts, be more sensitive to caffeine-related arousal effects [13]. Such factors may partly explain variability in reported associations between coffee consumption and depression across different populations.

Taken together, these vulnerability profiles illustrate why coffee and caffeine cannot be meaningfully framed as uniformly beneficial or harmful in depression. Their effects depend on individual sensitivity, comorbid anxiety, sleep vulnerability, hormonal factors and behavioural context. From a clinical perspective, this implies that caffeine use should be considered in terms of individual trade-offs between improved daytime functioning and potential exacerbation of sleep or anxiety symptoms.

## 8. Integrative Perspective: Caffeine as a Context-Dependent Modulator of Depressive Symptoms

The evidence reviewed above suggests that the relationship between coffee, caffeine and depression cannot be adequately captured by a simple protective or harmful model. Much of the epidemiological literature has focused on inverse associations between habitual coffee consumption and risk of depressive disorder, often interpreted as indicating a beneficial effect. However, such interpretations may be overly reductionist, as they collapse multiple symptom-level and behavioural processes into a single diagnostic outcome [8].

Available evidence indicates that caffeine mainly influences momentary symptom expression—particularly fatigue and alertness—without clear evidence that it alters the long-term course of depressive disorders. Its acute pharmacological action on adenosine signalling increases arousal and psychomotor activation, which can temporarily alleviate fatigue, low energy and cognitive slowing—symptoms that are central to many depressive presentations [28]. These short-term improvements are functionally meaningful, yet they do not necessarily imply sustained changes in core mood pathology.

On the other hand, the same mechanisms that enhance alertness may destabilise sleep–wake regulation and increase anxiety-related arousal, particularly in individuals with heightened vulnerability. Improvements in daytime alertness may therefore occur alongside poorer nocturnal sleep, which can destabilise mood regulation over time [32]. Over longer periods, such trade-offs may contribute to symptom variability rather than consistent clinical benefit, even when caffeine use is subjectively perceived as helpful.

Tolerance, habituation and withdrawal further complicate this picture. Regular caffeine consumption may prevent withdrawal-related fatigue and low mood, thereby creating the impression of mood improvement relative to abstinence. In this sense, some of the apparent benefits attributed to caffeine may reflect stabilisation of arousal states rather than true enhancement beyond an individual’s baseline functioning [20,30].

The anti-inflammatory and antioxidant properties of the coffee matrix add another layer, but their relevance is likely indirect and cumulative rather than acutely mood-enhancing. While such mechanisms may contribute to long-term modulation of physiological vulnerability, current evidence does not support a robust or specific antidepressant effect mediated through inflammatory pathways [39]. Their role should therefore be considered complementary to, rather than primary over, arousal- and sleep-related mechanisms.

Taken together, these converging lines of evidence suggest that coffee and caffeine operate within a complex behavioural and physiological system that regulates energy, sleep and emotional reactivity. Framing caffeine as either protective or detrimental in depression risks oversimplifying this system and obscuring the fact that its effects are conditional on individual sensitivity, habitual use patterns and baseline symptom profile. A more nuanced interpretation is that caffeine modifies how depressive symptoms are experienced and managed in daily life, without necessarily altering the fundamental course of the disorder.

This perspective does not negate epidemiological observations of inverse associations between coffee consumption and depression. Rather, it highlights that such associations may emerge from a combination of mechanisms, including functional self-regulation of fatigue, lifestyle correlates of coffee consumption, withdrawal-related symptom reversal and individual differences in arousal regulation. Ignoring these interacting processes may lead to overgeneralised conclusions about benefit and to insufficiently critical interpretation of nutripsychiatric findings.

## 9. Discussion

The present review aimed to reinterpret the role of coffee and caffeine in depression from a symptom-level and clinically contextual perspective rather than as a putative antidepressant exposure. For this reason, observational and symptom-focused human studies were treated as the primary evidentiary core of this review, whereas interventional studies were considered complementary but limited by substantial heterogeneity in compounds tested, dosing, duration and clinical populations. This heterogeneity makes it difficult to draw consistent conclusions regarding their clinical relevance, particularly in relation to specific depressive symptom domains. Evidence from experimental and observational studies converges on a limited but consistent finding: caffeine can transiently improve alertness and task engagement in individuals with fatigue-related depressive symptoms. Although coffee is embedded in broader dietary and lifestyle contexts, the present review focuses on coffee/caffeine exposure per se rather than on a predefined dietary pattern. The evidence reviewed in this work largely supports the initial assumption that caffeine operates primarily as a context-dependent modulator of symptom expression, although it also highlights important boundary conditions that refine this perspective.

From a clinical standpoint, the most plausible domain of benefit concerns fatigue, low energy and psychomotor slowing. These symptoms are among the most persistent and disabling features of depression and often remain even when core mood symptoms partially remit. Acute caffeine intake may transiently improve alertness, task initiation and perceived energy, which can facilitate engagement in daily activities and social functioning [27,29]. For some patients, especially those with pronounced anergia or hypersomnia, such functional effects may be subjectively meaningful and may support behavioural activation in the short term.

However, these potential benefits are inseparable from clinically relevant trade-offs. The same pharmacological mechanisms that increase arousal may also exacerbate anxiety, inner tension and sleep disruption, particularly in individuals with heightened anxiety sensitivity or baseline sleep vulnerability [32,43]. In such cases, improvements in daytime energy may coexist with deterioration in nocturnal sleep and emotional regulation, potentially contributing to symptom instability rather than sustained improvement.

Tolerance and withdrawal dynamics further complicate clinical interpretation. Regular caffeine consumption may prevent withdrawal-related fatigue, irritability and low mood, which overlap with core depressive symptoms [43]. Consequently, perceived mood benefits following caffeine intake may partly reflect withdrawal reversal rather than true enhancement of baseline affective functioning [30]. Clinicians should therefore be cautious in interpreting self-reported improvements in mood or energy that are tightly linked to habitual caffeine use patterns.

The concept of self-medication is particularly relevant in this context. Patients experiencing lethargy, excessive daytime sleepiness or reduced motivation may intentionally use caffeine to counteract these symptoms. While such strategies may be adaptive in maintaining short-term functioning, they can also obscure the underlying severity of depressive symptoms and contribute to delayed recognition of sleep disturbance or comorbid anxiety as maintaining factors. This suggests that caffeine use in depression may often reflect compensatory behavioural regulation rather than a direct therapeutic effect on mood pathology.

Importantly, the available evidence does not support framing coffee or caffeine as antidepressant interventions in a clinical sense. Observational findings showing inverse associations between coffee consumption and depression risk [9] should be interpreted cautiously in clinical contexts. Such associations may partly reflect reverse causality, functional lifestyle correlates and individual patterns of coping with fatigue rather than causal protection against depressive disorder.

At the same time, a strictly negative interpretation would also be misleading. For many individuals with depression, moderate caffeine use embedded in stable daily routines may support daytime functioning without substantial adverse effects. The clinical relevance, therefore, lies less in universal recommendations and more in individualised assessment of benefits and costs, including effects on sleep, anxiety and overall symptom stability. This assessment should also take into account broader contextual factors such as occupational rhythm, social functioning, smoking, alcohol use, general dietary pattern and other environmental influences that may shape both habitual coffee use and depressive symptom expression.

These considerations highlight the importance of moving beyond binary narratives of caffeine as either beneficial or harmful in depression. A more clinically useful perspective is to conceptualise caffeine as an acute arousal-modulating factor, capable of both alleviating fatigue and exacerbating arousal-related symptoms depending on dose, timing, habitual use and individual vulnerability profile. Such a framework may help clinicians engage in more nuanced discussions with patients regarding caffeine consumption, focusing on functional outcomes and trade-offs rather than on simplistic assumptions of benefit or harm.

Overall, the present synthesis suggests that caffeine’s role in depression is best understood not in terms of disease modification, but rather as short-term regulation of energy, alertness and behavioural activation within complex daily contexts. Recognising this distinction may improve both clinical interpretation and future research design by encouraging more symptom-level, temporally sensitive and context-aware approaches to studying dietary psychoactive exposures in mental health. Additional factors such as environmental exposures, broader dietary patterns and the gut microbiome may also influence the relationship between caffeine use and mental health, but these were beyond the scope of the present review. Importantly, this perspective does not support the interpretation of coffee or caffeine as therapeutic interventions for mental disorders, but instead frames them as factors that may transiently influence symptom expression, often in a context-dependent manner.

## 10. Conclusions and Future Directions

Existing studies do not provide sufficient evidence to treat coffee or caffeine as clinically meaningful antidepressant interventions. Rather, available data are more consistent with a role for caffeine as a behaviorally embedded stimulant affecting daily functioning, particularly in domains such as fatigue, psychomotor activation and daytime alertness. These effects are primarily acute and state-dependent, and are strongly influenced by sleep–wake regulation, habitual use patterns and individual sensitivity to stimulation.

Inverse associations between habitual coffee consumption and depression risk reported in cohort studies should therefore be interpreted cautiously. Reverse causality, residual confounding, withdrawal-related symptom reversal and unmeasured sleep disturbance may substantially contribute to these observations and limit causal inference.

Clinically, caffeine may have a dual role. Moderate use may support short-term functioning in individuals with prominent fatigue, whereas in those with anxiety, sleep vulnerability or mood instability, it may exacerbate arousal and contribute to symptom fluctuation. This heterogeneity highlights the need for individualised interpretation rather than generalised recommendations.

Future research should prioritise symptom-level and longitudinal designs that account for dose, timing of intake, sleep–wake parameters and habitual use patterns. Distinguishing between withdrawal reversal and true symptom modification will be particularly important for avoiding overestimation of beneficial effects.

Overall, caffeine is better understood as a behavioural and physiological modulator of energy and arousal in depression rather than as a factor modifying the underlying course of the disorder.

## 11. Limitations

This review has several limitations. First, as a narrative synthesis, it does not provide pooled quantitative estimates and may be influenced by heterogeneity in study designs, populations and outcome definitions. Second, the proposed integrative model relies on combining epidemiological, experimental and mechanistic evidence, which involves a degree of inferential extrapolation, particularly given the limited number of studies conducted directly in clinically diagnosed depressive populations. Third, key moderators such as sleep disturbance, anxiety sensitivity and withdrawal-related effects are not consistently assessed in large observational studies, limiting empirical verification of the pathways discussed. Fourth, reverse causality remains a significant concern, as individuals with early fatigue or hypersomnia may increase caffeine intake as a compensatory strategy. Finally, the clinical implications should be interpreted cautiously, as the evidence base is indirect and heterogeneous. Individual variability in sensitivity, comorbid anxiety and habitual use patterns may substantially modify responses to caffeine, restricting the generalisability of the conclusions.

## Data Availability

AI-assisted tools (including Canva AI) were used solely for the preparation of illustrative graphical materials and for minor language editing to improve the clarity of the English text. All scientific content, including the literature search, study selection, interpretation of evidence, data analysis and formulation of conclusions, was performed exclusively by the authors without the use of AI tools.
